# Optimizing Peafowl (*Pavo cristatus*) Fertility and Chick Growth With Mealworm‐Based Feed Supplementation

**DOI:** 10.1002/vms3.70767

**Published:** 2026-01-14

**Authors:** Sar Zamin Khan, Hanan Al‐Khalaifah, Haris Khan, Rifat Ullah Khan, Shabana Naz, Ala Abudabos, Ibrahim A. Alhidary

**Affiliations:** ^1^ Department of Poultry Science Faculty of Animal Husbandry and Veterinary Sciences The University of Agriculture Peshawar Pakistan; ^2^ Environment and Life Sciences Research Center Kuwait Institute for Scientific Research Safat Kuwait; ^3^ Physiology Lab College of Veterinary Sciences, Faculty of Animal Husbandry and Veterinary Sciences, The University of Agriculture Peshawar Pakistan; ^4^ Department of Zoology Government College University Faisalabad Pakistan; ^5^ Department of Food and Animal Sciences College of Agriculture, Tennessee State University Nashville USA; ^6^ Department of Animal Production College of Food and Agriculture Science King Saud University Riyadh Saudi Arabia

**Keywords:** chick growth performance, hatchability, insect protein supplementation, mealworm meal, peafowl reproduction

## Abstract

This study evaluated the effects of mealworm meal premix supplementation on the reproductive performance of adult peafowl and the growth and survivability of their chicks. Mealworms were oven‐dried, de‐oiled, ground and mixed with fish waste powder (150 g/kg) to prepare a nutritional premix. A total of 120 adult peafowl were allocated into four groups: control (0 g/kg) and three treatment groups receiving 20, 40 and 60 mg/kg of mealworm premix, respectively. Birds were monitored for feed intake, egg production, fertility, hatchability and feed conversion ratio (FCR). Hatched chicks were reared for 4 weeks under controlled brooding conditions, and their feed intake, weight gain and survivability were recorded. Results indicated a significant improvement (*p*  ≤  0.05) in FCR, chick weight, feed intake (for the first 2 weeks) and weekly weight gain in the supplemented groups, particularly at 60 mg/kg inclusion. Fertility and hatchability percentages were consistently higher in treated groups, though not always statistically significant. Chick livability improved markedly during early brooding, with Groups C and D achieving 100% survival by Week 1. The findings suggest that mealworm meal premix enhances reproductive efficiency in breeding peafowl and supports early chick development and survival, highlighting its potential as a sustainable and functional feed additive in avian species.

## Graphical abstract

Mealworm meal premix supplementation in breeding peafowl diets improved feed conversion, chick growth and early survivability, particularly at higher inclusion levels. The premix supported reproductive performance and chick vitality without adverse effects, indicating its potential as a sustainable functional feed additive.

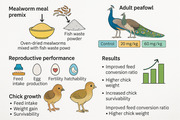

## 1 Introduction

The Indian peafowl (*Pavo cristatus*), a nationally protected species in South Asia, holds ecological, aesthetic and genetic significance in both conservation and ornamental aviculture programmes. Despite increasing interest in its captive breeding, there is a paucity of scientific data addressing its specific nutritional needs—particularly in relation to reproductive performance and early chick development under managed conditions. Unlike commercial poultry species, whose dietary responses are well‐characterized, peafowl remain underrepresented in applied nutritional research, creating a critical knowledge gap in optimizing feeding strategies for this species (Chebo et al. 2024). While mealworm supplementation has been extensively studied in chickens with known effects on egg production, immunity and growth, similar data are completely lacking for ornamental birds like peafowl, whose reproductive biology and dietary responses may differ substantially.

Insect‐based proteins, particularly mealworm (*Tenebrio molitor*) meal, have gained attention as sustainable alternatives to conventional protein sources like fish meal and soybean meal due to their high protein content, superior amino acid profile and lower environmental footprint (Gkinali et al. 2022; Bovera et al. 2015. Studies in broilers and laying hens have demonstrated the beneficial effects of mealworm supplementation on growth, immunity and nutrient utilization (De Marco et al. 2015; Sedgh‐Gooya et al. 2021; Al‐Khalaifah et al. 2025a, b). However, these findings cannot be directly extrapolated to peafowl due to species‐specific differences in physiology, metabolism and dietary behaviour.

To date, no empirical studies have evaluated the reproductive or productive responses of peafowl to dietary mealworm inclusion. This represents a clear gap, especially considering the reproductive challenges frequently encountered in captive peafowl breeding, such as low fertility, poor hatchability and high chick mortality. Improving reproductive efficiency in captive peafowl is not only vital for conservation but also for the sustainability of ornamental aviculture, wildlife tourism and private breeding programmes where reproductive failure poses economic risks.

This study was therefore designed to investigate the effects of graded levels of mealworm meal premix on fertility, hatchability, hatch weight, feed intake and livability in breeding Indian peafowl. By doing so, it aims to generate species‐specific data that can inform feeding strategies and support sustainable captive management practices for this iconic bird.

## 2 Materials and Methods

### 2.1 Preparation of Mealworm Meal Premix

Mealworms were collected and oven‐dried for 2–4 h at 60°C–80°C in the Mealworm Laboratory established under the PSF‐funded project PSF/NSLP/KP‐UAP (709) at the University of Agriculture, Peshawar. The dried worms were then de‐oiled using a cold press oil extraction machine (Model: AG‐OIL‐01, AgriPro Industries, Germany) and finely ground using a laboratory grinder (Model: GR‐150, Hanchen Scientific Instruments, China) to obtain mealworm powder. This powder was subsequently analysed for proximate composition. Fish waste was procured from local fish markets and restaurants, sun‐dried and ground into powder at the Department of Poultry Science. The fish waste powder was incorporated into the mealworm powder at a ratio of 150 g/kg to formulate the final premix. The inclusion rate of fish meal was kept constant across all diets to avoid confounding effects.

### 2.2 Proximate and Chemical Composition of Superworm

Proximate analysis of dried mealworms was performed following the standard procedures outlined by the Association of Official Analytical Chemists (AOAC 2000). Metabolizable energy (kcal/kg) was calculated using the formula: (g of crude protein × 4.0) + (g of crude fat × 9.0) + (g of nitrogen‐free extract × 4.0). The amino acid and mineral contents of mealworm samples were determined according to AOAC protocols (AOAC 1990). Amino acids were quantified using the PICO TAG method (PICO TAG amino acid analyser, Model: 9200, Waters Corporation, USA) with minor modifications, based on peak area measurements generated from standard curves using a PICO TAG amino acid analyser. Water‐ and fat‐soluble vitamins were analysed by high‐performance liquid chromatography (HPLC) following the method described by Mateeva et al. (2023). All equipment was routinely calibrated before use.

### 2.3 Birds Housing and Grouping

A total of 120 adult peafowl (30♂ + 90♀) of almost similar average body weight (2.8  ±  0.2 kg) and age (2 years) were used for this study. The sample size was determined based on previous studies on reproductive performance in birds and allowed for detecting moderate effect sizes with ≥ 80% power and *α* = 0.05. All peafowl were assigned to four experimental groups (A, B, C and D), and housed in an open caging system measuring 10 × 10 × 12 feet with a 5 × 5 × 4 feet covered area, having a sex ratio (1♂: 3♀) for each experimental group. Birds in Group A were provided with 150 g of commercial broiler breeder ration, while each bird in Groups B, C and D was provided with the same amount of commercial broiler breeder ration supplemented with mealworm meal premix at the rate of 20, 40 and 60 g/kg of ration, respectively. The diets were formulated to be isocaloric and isonitrogenous (Tables 1 and 2).

**TABLE 1:** Corn‐soybean meal‐based basal diet with mealworm meal premix supplementation.

Ingredient	Control (0 mg/kg)	20 mg/kg	40 mg/kg	60 mg/kg
Corn	57.0	56.5	55.8	55.0
Soybean meal (44%)	31.0	29.0	27.5	26.0
Mealworm premix	0.0	3.0	3.0	3.0
Fish meal (optional)	3.0	2.0	4.0	6.0
Vegetable oil	3.8.0	3.6	3.4	3.2
Dicalcium phosphate	1.2.0	1.2	1.20	1.20
Limestone	1.45	1.45	1.45	1.45
Vitamin–mineral premix^1^	0.5	0.30	0.30	0.30
DL‐Methionine	0.18	0.18	0.50	0.5
L‐Lysine HCl	0.27	0.27	0.18	0.18
Chemical analysis				
Crude protein, %	18.0	18.0	18.0	18.0
Metabolizable energy, kcal/kg	2800	2800	2800	2800
Methionine, %	0.45	0.45	0.45	0.45
Lysine, %	0.90	0.90	0.90	0.90
Calcium, %	34	34.0	34.0	34.0

^1^ Provided per kg of diet: vitamin A, 8000 IU; vitamin D_3_,2000 IU; vitamin E,15 IU; vitamin K_3_, 1.5 mg; thiamine, 2 mg; riboflavin, 5 mg; pyridoxine, 5 mg; vitamin B_12_, 0.02 mg; folic acid, 0.7 mg; nicotinic acid, 40 mg; pantothenic acid, 12 mg; biotin, 0.2 mg.

^b^Provided per kg of diet: copper, 10 mg (as copper sulphate); iron, 90 mg (as ferrous sulphate); manganese, 100 mg (as manganese sulphate); zinc, 100 mg (as zinc sulphate); selenium, 0.3 mg (as sodium selenite); iodine, 0.5 mg (as calcium iodate).

**TABLE 2:** Proximate and chemical analysis of de‐oiled MWM premix.

Proximate analysis	Amino acid (mg/kg)	Mineral profile (mg/kg)	Vitamin profile
Moisture 06%	Isoleucine 14.00	Calcium 390	Vitamin A (IU/kg) 3400
Dry matter 94%	Leucine 19.00	Phosphorus 2900	Vitamin D_2_ (IU/kg) 534
Crude protein 80 %	Lysine 14.00	Magnesium 692	Vitamin D_3_ (IU/kg) < 40
Crude fat 10%	Methionine 6.00	Sodium 435	Vitamin E (IU/kg) 163.0
Crude Fibre 3.5%	Phenylalanine 8.00	Potassium 3388	Vitamin K (mg/kg) < 50
Crude ash 6.5%	Proline 13.00	Chloride 1765	Vitamin C (mg/kg) 100.0
M.E. 2400 (kcal/kg)	Serine 10.00	Iron 35	Thiamine (mg/kg) 1.7
	Threonine 8.00	Zinc 49	Riboflavin (mg/kg) 11.4
	Tryptophan 4.00	Copper 5.33	Pantothenic acid (mg/kg) 7.3
	Tyrosine 13.50	Manganese 3.40	Niacin (mg/kg) 35.3
	Valine 13.00	Iodine 0.12	Pyridoxine (mg/kg) 3.70
	Taurine 0.13	Selenium 0.225	Folic acid (mg/kg) 0.65
			Biotin (mg/kg) 0.38
			Vitamin B_12_ (µg/kg) 9.9
			Choline (mg/kg) 1246
			Inositol (mg/kg) 225

### 2.4 Growth Performance

Feed remnants were weighed and removed from each feeding pot to determine feed intake. Total and average feed intake per peafowl group were calculated weekly. Beginning from the onset of egg laying, daily egg production was recorded, and eggs from each group were labelled with relevant identification details. Weekly egg production per peafowl group was calculated accordingly. The average egg weight (g) was measured weekly using a digital precision scale (Model: EK‐300i, A&D Company, Japan; accuracy:  ± 0.01 g). These data were used to determine the total egg mass produced by each group, which was then utilized to calculate the feed conversion ratio (FCR), using the formula:

$$\mathrm{FCR}=\mathrm{Feed}\;\mathrm{consumed}\left(g\right)/\mathrm{Egg}\;\mathrm{mass}\;\mathrm{produced}\left(\mathrm{kg}\right)$$

Collected eggs were placed in trays and stored in a room maintained at 15°C–18°C with a relative humidity of 75%–80% until incubation.

### 2.5 Hatchery Conditions, and Calculation of Hatchability and Fertility

The eggs were transferred to an incubator for incubating at 37.7°C and 65% moisture for 29 days in the development section, and at 37.5°C and 90% moisture for the last 3 days (Hafeez et al. 2024). The weight of newly hatched chicks was measured using a digital weighing balance (Model: BL2000, Shimadzu, Japan; precision:  ± 0.01 g). The fertility and hatchability rates were calculated by using the following formulas (Hafeez et al. 2024):

Fertility=Numberoffertileeggs/Totalnumberofeggsset;

Hatchability=Numberofchickhatched/Totaleggsset

### 2.6 Rearing of Newly Hatched Chicks

Newly hatched peafowl chicks (120) were randomly assigned to four experimental groups (A, B, C and D) based on their parental origin. Each group was provided a standard commercial broiler starter ration, supplemented with mealworm meal premix at inclusion rates of 0, 20, 40 and 60 g/kg of feed, respectively. The chicks were reared under controlled brooding conditions for a period of 4 weeks. During the first week, the brooding temperature was maintained at 35  ±  1°C, and then gradually reduced by 2–3°C each subsequent week until it reached 26–28°C by the end of the fourth week. Continuous lighting (24 h) was provided during the first 3 days to encourage feeding and adaptation. From Day 4 onwards, a 20‐h light and 4‐h dark cycle was maintained to support optimal growth and development. All chicks were monitored twice daily for signs of illness or injury. Mortality, if any, was recorded, and dead birds were removed promptly for post‐mortem evaluation. No chicks were excluded from the analysis unless death occurred. Chicks were housed in clean, disinfected pens equipped with appropriate bedding, feeders and drinkers, and were monitored daily for health and welfare. Clean, fresh water and feed were offered ad libitum throughout the trial. Performance parameters including weekly body weight gain, total feed intake and survivability (livability percentage) were recorded and analysed for each group over the 4‐week rearing period.

### 2.7 Statistical Analysis

Data on the production performance of breeding peafowl and survival of newly hatched chicks were collected within a specific duration for each supplemented group. One‐way ANOVA was set to analyse the collected data using statistical software (Statistix 8.1), and LSD test was used to link the differences among all selected parameters.

## 3 Results

The FCR of breeding peafowl supplemented with different levels of mealworm meal premix is presented in Table 3. During the first 2 weeks, there were **no** significant differences (*p* > 0.05) observed in FCR among all experimental groups, suggesting a uniform initial response to the diets. However, from Week 3 onwards, significant (*p* ≤ 0.05) improvements in FCR were observed in the groups receiving mealworm meal, particularly at higher inclusion levels. In Week 3, Group D (60 mg/kg) showed a significantly better FCR (1.02  ±  0.01) compared to all other groups, while Groups A, B and C showed no significant difference among themselves. A gradual and consistent improvement in FCR was noted in the supplemented groups across the subsequent weeks. By Week 4, the FCR values significantly differed (*p* = 0.04), with the lowest FCR recorded in Group D (0.81  ±  0.01), followed by Group C (1.02  ±  0.01), Group B (1.36  ±  0.02) and the highest in the control group (2.06  ±  0.01). This trend continued through Weeks 5–10, where the highest level of mealworm meal supplementation (60 mg/kg) consistently yielded the best FCR values, with statistically significant differences (*p* ≤ 0.05) compared to lower inclusion levels and the control. Notably, in Week 6, Group D exhibited the most efficient feed conversion (0.67  ±  0.03), which was significantly better than Groups A (1.38  ±  0.01), B (1.36  ±  0.04) and C (1.02  ±  0.01). By the final week (Week 10), the FCR of Group D (1.01  ±  0.04) remained significantly lower than all other groups, indicating sustained efficiency.

**TABLE 3:** FCR of breeding peafowl supplemented with different levels of mealworm meal premix.

	Groups				
Weeks	A	B	C	D	*p* value
01 02	2.25 ± 0.02 2.15 ± 0.01	2.23 ± 0.01 2.12 ± 0.01	2.28 ± 0.04 2.10 ± 0.01	2.31 ± 0.09 2.13 ± 0.02	0.09 0.15
03	2.07 ± 0.01^a^	2.04 ± 0.02^a^	2.04 ± 0.02^a^	1.02 ± 0.01^b^	0.03
04	2.06 ± 0.01^a^	1.36 ± 0.02^b^	1.02 ± 0.01^c^	0.81 ± 0.01^d^	0.04
05	1.38 ± 0.02^a^	1.37 ± 0.01^a^	1.36 ± 0.03^a^	0.81 ± 0.02^b^	0.05
06	1.38 ± 0.01^a^	1.36 ± 0.04^a^	1.02 ± 0.01^b^	0.67 ± 0.03^c^	0.02
07	2.0 ± 0.06^a^	1.36 ± 0.03^b^	0.81 ± 0.02^c^	0.67 ± 0.01^c^	0.01
08	1.38 ± 0.03^a^	1.36 ± 0.02^a^	1.02 ± 0.01^b^	0.81 ± 0.02^c^	0.02
09	2.06 ± 0.02^a^	1.36 ± 0.04^b^	1.02 ± 0.03^c^	1.01 ± 0.01^c^	0.02
10	2.0 ± 0.06^a^	2.04 ± 0.02^a^	1.36 ± 0.04^b^	1.01 ± 0.04^c^	0.04

*Note*: Means within a row with different superscripts indicate significant difference at (*p* ≤ 0.05).

Abbreviations: Group A, control; Group B, 20 mg/kg; Group C, 40 mg/kg; Group D, 60 mg/kg.

The effect of mealworm meal premix supplementation on the fertility and hatchability (%) of breeding peafowl is summarized in Tables 4 and 5, respectively. Throughout the entire observation period, there was no statistically significant difference in fertility and hatchability percentages among the treatment groups (*p* > 0.05), indicating that supplementation with different levels of worm meal premix had no significant effect on the fertility and hatchability of breeding peafowl.

**TABLE 4:** Fertility (%) of breeding peafowl supplemented with different levels of worm meal premix.

	Groups				
Weeks	A	B	C	D	*p* value
01	00	00	00	00	0.00
02	00	00	00	00	0.00
03	82 ± 3.2	85 ± 7.1	86 ± 3.5	85 ± 3.4	0.07
04	82 ± 3.5	83 ± 8.4	83 ± 5.6	84 ± 5.6	0.06
05	85 ± 5.1	85 ± 6.2	86 ± 4.2	85 ± 5.2	0.10
06	84 ± 4.9	85 ± 5.3	86 ± 4.5	85 ± 3.5	0.15
07	84 ± 3.4	85 ± 7.1	86 ± 6.7	85 ± 4.5	0.17
08	84 ± 2.6	85 ± 5.5	86 ± 4.5	85 ± 6.5	0.21
09	84 ± 5.1	85 ± 7.3	86 ± 8.5	85 ± 5.5	0.19
10	84 ± 2.9	85 ± 4.6	86 ± 4.3	85 ± 3.5	0.12
11	00	00	00	00	0.00
12	00	00	00	00	0.00

Abbreviations: Group A, control; Group B, 20 mg/kg; Group C, 40 mg/kg; Group D, 60 mg/kg.

**TABLE 5:** Hatchability (%) of breeding peafowl supplemented with different levels of worm meal premix.

	Groups				
Weeks	A	B	C	D	*p* value
01	00	00	00	00	0.00
02	00	00	00	00	0.00
03	63.1 ± 2.1	66	65 ± 0.00	66 ± 0.00	0.10
04	65.3 ± 4.4	65.3 ± 8.1	66 ± 0.00	67 ± 0.00	0.14
05	65.4 ± 5.2	65 ± 5.4	66 ± 0.00	67 ± 0.00	0.11
06	65.5 ± 1	65 ± 3.1	65 ± 0.00	69 ± 0.00	0.18
07	65.6 ± 6.2	65 ± 6.3	65 ± 0.00	67 ± 0.00	0.10
08	65 ± 3	65 ± 5.3	66 ± 0.00	68 ± 0.00	0.14
09	65 ± 4.6	65 ± 6.2	66 ± 0.00	68 ± 0.00	0.12
10	65 ± 6.4	65 ± 1.9	66 ± 0.00	67 ± 0.00	0.11

*Note*: Means within a row with different superscripts indicate significant difference at (*p* ≤ 0.05).

Abbreviations: Group A, control; Group B, 20 mg/kg; Group C, 40 mg/kg; Group D, 60 mg/kg.

The chick hatch weight of peafowl in response to varying levels of mealworm meal premix supplementation is shown in Table 6.

**TABLE 6:** Chick's weight (g) of peafowl during hatch supplemented with different levels of worm meal premix.

	Groups				
Weeks	A	B	C	D	*p* value
01	0.0 ± 0.0	0.0 ± 0.0	0.0 ± 0.0	0.0 ± 0.0	0
02	0.0 ± 0.0	0.0 ± 0.0	0.0 ± 0.0	0.0 ± 0.0	0
03	38.9 ± 1.9	41 ± 2.8	41 ± 3.5	42 ± 2.8	0.11
04	40.1 ± 2.1	42 ± 2.5	42 ± 5.1	43 ± 3.4	0.06
05	40.1 ± 3.4^b^	45 ± 4.3^ab^	46 ± 2.8^a^	47 ± 6.1^a^	0.05
06	40.2 ± 2.3^c^	43 ± 5.1^b^	45 ± 5.1^a^	49 ± 5.6^a^	0.01
07	42.1 ± 1.9^c^	43 ± 3.5^bc^	45 ± 5.8^b^	49 ± 3.5^a^	0.01
08	42.1 ± 2.8^c^	43 ± 2.5^bc^	45 ± 4.5^b^	49 ± 6.9^a^	0.02
09	40.2 ± 3.1^c^	43 ± 1.7^bc^	45 ± 3.4^b^	49 ± 4.5^a^	0.00
10	40.8 ± 2.6^c^	43 ± 3.5^bc^	45 ± 2.9^b^	49 ± 6.1^a^	0.01

*Note*: Means within a row with different superscripts indicate significant difference at (*p* ≤ 0.05).

Abbreviations: Group A, control; Group B, 20 mg/kg; Group C, 40 mg/kg; Group D, 60 mg/kg.

No chicks hatched during Weeks 1 and 2 across all treatment groups, with weights recorded as 0.0  ±  0.0 g. From Week 3 onwards, chicks began to hatch, and chick weight increased progressively across the groups. In Weeks 3 and 4, chick weights ranged from 38.9 to 43 g across Groups A to D, with no statistically significant differences observed (*p* > 0.05). However, beginning in Week 5, statistically significant differences emerged (*p* ≤ 0.05). From Weeks 5 to 10, Group D (60 mg/kg worm meal) consistently produced significantly heavier chicks (ranging from 47 to 49 g), followed by Group C (40 mg/kg) and Group B (20 mg/kg). Group A (control) consistently had the lowest chick weights throughout this period. The most pronounced differences were observed in Weeks 6–10, where Group D's chicks were significantly heavier (*p* ≤ 0.01 to *p* = 0.00) compared to all other groups.

The feed intake of peafowl chicks supplemented with different levels of mealworm meal premix over a 4‐week period is summarized in Table 7. Significant differences in feed intake were observed among the groups, particularly during the early weeks of brooding. In Week 1, chicks in Groups C (780 g) and D (785 g) consumed significantly more feed compared to Groups A and B (both 660 g), with a highly significant difference (*p* = 0.00). This suggests a stimulatory effect of mealworm meal at higher inclusion levels on early feed intake. During Week 2, the trend persisted, with Group D exhibiting the highest feed intake (1154 g), significantly greater than Group A (923 g) and Group B (950 g), while Group C (956 g) showed similar intake to Group B. The differences were statistically significant (*p* = 0.02). By Week 3, the differences became more pronounced. Groups B, C and D all recorded similar and significantly higher feed intake values (ranging from 1556 to 1567 g) compared to Group A (1360 g), with *p* = 0.00 indicating high statistical significance. In Week 4, the feed intake among groups was more comparable. Groups C and D showed the highest intake (2140–2143 g), followed by Groups B and A (2135 and 2134 g, respectively).

**TABLE 7:** Feed intake (g) of peafowl chicks (*n* = 10) supplemented with different levels of worm meal premix.

	Groups				
Weeks	A	B	C	D	*p* value
01	660 ± 9.3^b^	660 ± 13.5^b^	780 ± 6.1^a^	785 ± 8.5^a^	0.00
02	923 ± 11.40^c^	950 ± 14.7^b^	956 ± 5.30^b^	1154 ± 9.40^a^	0.02
03	1360 ± 12.5^b^	1567 ± 11.4^a^	1556 ± 12.5^a^	1560 ± 12.3^a^	0.001
04	2134 ± 9.1	2135 ± 8.9	2140 ± 11.1	2143 ± 11.2	0.06

*Note*: Means within a row with different superscripts indicate significant difference at (*p* ≤ 0.05).

Abbreviations: Group A, control; Group B, 20 mg/kg; Group C, 40 mg/kg; Group D, 60 mg/kg.

The weekly weight gain of peafowl chicks supplemented with increasing levels of mealworm meal premix is presented in Table 8. A consistent and statistically significant improvement in weight gain was observed in the supplemented groups compared to the control (Group A) over the 4‐week period. In Week 1, chicks in Group D (60 mg/kg) recorded the highest weight gain (510 g), followed closely by Group C (490 g), both significantly higher than Group B (455 g) and Group A (400 g), with a significant difference at *p* = 0.02. This indicates a positive initial response to higher levels of mealworm meal inclusion. During Week 2, the trend continued, with Group D again showing the greatest weight gain (623 g), followed by Group C (611 g), Group B (546 g) and Group A (480 g). All differences were statistically significant (*p* = 0.03), reinforcing the growth‐promoting potential of the premix. In Week 3, weight gain remained significantly higher in all supplemented groups compared to the control. Group D (745 g) achieved the highest gain, followed by Group C (723 g), Group B (711 g) and Group A (630 g), with a highly significant difference (*p* = 0.01). By Week 4, although the magnitude of differences narrowed slightly, Group D (812 g) still recorded the highest weight gain, significantly greater than Group C (801 g), while Groups B and A had similar but lower gains (790 and 786 g, respectively).

**TABLE 8:** Weight gain of peafowl chicks (*n* = 10) supplemented with different levels of worm meal premix.

	Groups				
Weeks	A	B	C	D	*p* value
01	400 ± 13.4^c^	455 ± 11.2^c^	490 ± 14.5^b^	510 ± 12.3^a^	0.02
02	480 ± 12.5^d^	546 ± 12.3^c^	611 ± 15.6^b^	623 ± 13.5^a^	0.03
03	630 ± 11.2^d^	711 ± 13.5^c^	723 ± 16.5^b^	745 ± 15.6^a^	0.01
04	786 ± 10.9^c^	790 ± 15.6^c^	801 ± 17.5^b^	812 ± 16.7^a^	0.05

*Note*: Means within a row with different superscripts indicate significant difference at (*p* ≤ 0.05).

Abbreviations: Group A, control; Group B, 20 mg/kg; Group C, 40 mg/kg; Group D, 60 mg/kg.

The livability (%) of peafowl chicks supplemented with different levels of mealworm meal premix is shown in Table 9. A clear positive effect on survivability was observed with increasing levels of supplementation during the first 3 weeks of brooding. In Week 1, the control group (Group A) recorded the lowest livability at 80%, significantly lower than Group B (90%) and both Groups C and D, which achieved 100% livability (*p* = 0.00). This indicates an immediate improvement in chick survival with even the lowest level of premix inclusion. By Week 2, Group D continued to maintain 100% livability, while Groups B and C showed 90%, and Group A remained at 80%, with statistically significant differences (*p* = 0.00). These results demonstrate the enhanced early survivability provided by mealworm supplementation. In Week 3, the livability of the control group increased slightly to 90%, while all supplemented groups (B, C and D) achieved 100% survival. The differences were still statistically significant (*p* = 0.05), reflecting the sustained benefit of the premix during the critical brooding phase. By Week 4, all groups reached 100% livability, indicating that once chicks passed the initial brooding period, survival stabilized across treatments (*p* = 0.99).

**TABLE 9:** Livability (%) of peafowl chicks (*n* = 10) supplemented with different levels of worm meal premix.

	Groups				
Weeks	A	B	C	D	*p* value
01	80 ± 2.3^c^	90 ± 4.1^b^	100 ± 4.5^a^	100 ± 6.7^a^	0.00
02	80 ± 4.3^c^	90 ± 3.5^b^	90 ± 5.4^b^	100 ± 7.6^a^	0.00
03	90 ± 5.4^b^	100 ± 6.2^a^	100 ± 3.8^a^	100 ± 4.5^a^	0.05
04	100 ± 4.5^a^	100 ± 5.6^a^	100 ± 5.6^a^	100 ± 3.2^a^	0.99

*Note*: Means within a row with different superscripts indicate significant difference at (*p* ≤ 0.05).

Abbreviations: Group A, control; Group B, 20 mg/kg; Group C, 40 mg/kg; Group D, 60 mg/kg.

## 4 Discussion

In the current study, breeding peafowl supplemented with 60 mg/kg mealworm meal premix (Group D) consistently showed the lowest FCRs, particularly from Weeks 4 to 10 where values remained below 1.02, indicating significantly improved nutrient utilization and digestive efficiency compared to the control and lower supplementation groups. These findings are in agreement with previous studies that have highlighted the nutritional and functional benefits of insect‐based proteins, particularly *T. molitor* (mealworms), in poultry diets. Mealworms are rich in high‐quality protein, essential amino acids, lipids, vitamins and minerals, and their inclusion has been shown to improve feed efficiency in broilers (Bovera et al. 2016; Dabbou et al. 2018). The low FCR observed in Group D may be attributed to the high digestibility and favourable amino acid profile of mealworm protein, which enhances nutrient absorption and minimizes feed wastage.

However, the study did not include direct measurements of digestive enzyme activity or gut morphology; thus, the improved FCR must be interpreted as a functional outcome rather than evidence of enhanced gut physiology. Although previous studies suggest that insect‐derived bioactives such as chitin, antimicrobial peptides and lauric acid may contribute to gut health (Gasco et al. 2020; de Carvalho et al. 2019), our results do not confirm such mechanisms, as no microbiological or histological data were collected.

Furthermore, studies in quails and chickens have reported that insect meal inclusion at moderate levels (up to 10% of diet) significantly improves FCR and growth performance without adverse effects (Schiavone et al. 2017). In the present study, the 60 mg/kg inclusion level likely provided a balance between nutritional enrichment and palatability, avoiding potential negative effects of excessive chitin or fat content. Insect meals, especially those derived from *T. molitor* (mealworms), are rich in high‐quality protein, essential amino acids (e.g., lysine and methionine), vitamins and trace minerals, all of which are critical for reproductive function in birds (Bovera et al. 2015).

The observed improvements in chick hatch weight of peafowl following dietary supplementation with mealworm meal premix are consistent with findings in poultry nutrition research, where insect‐based proteins have been linked to enhanced embryonic development and chick vitality (Kere et al. 2023). The consistent increase in hatch weight across the supplemented groups—especially in Group D (60 mg/kg)—suggests that the mealworm meal provided a more balanced and bioavailable nutrient profile that supports fetal growth. Mealworm meal is a rich source of high‐quality protein, essential amino acids and biologically active compounds, which are vital during embryogenesis. According to Bovera et al. (2015), inclusion of *T. molitor* in poultry diets improved chick quality due to its superior amino acid profile compared to conventional feed ingredients. Increased chick hatch weights in the supplemented groups, particularly at higher inclusion levels, may also be attributed to the enhanced energy and lipid content of the mealworm meal. Mealworms are known to contain high levels of unsaturated fatty acids—particularly oleic and linoleic acids—which are essential for yolk lipid metabolism and fetal tissue growth (Schiavone et al. 2017; Sparks 2006).

Nevertheless, the study did not include biochemical assays of egg yolk composition or maternal serum, so nutrient transfer into the egg remains an inferred mechanism rather than a validated one. Future studies should assess egg nutrient content to confirm such transference. Furthermore, mealworm meal contains bioactive peptides and chitin, which have been reported to enhance gut health and immune development in chicks (Gasco et al. 2020). Although these mechanisms are well‐documented in broilers, our study did not investigate gut morphology or immune markers in chicks; therefore, we avoid drawing mechanistic conclusions regarding these outcomes in peafowl. Improved maternal gut health and nutrient absorption due to insect meal supplementation may result in higher nutrient deposition in eggs, supporting better embryo growth. As Marono et al. (2017) indicated, insect‐derived feed ingredients improved not only growth performance in broilers but also reproductive output and chick quality in breeders. Comparable studies in quail and broiler breeders have shown that insect meal supplementation led to heavier chicks at hatch, improved yolk utilization and better immune competence (Loponte et al. 2017; Sogari et al. 2019). These results are reflected in the present study, where increased chick weights in the treatment groups were both statistically significant and biologically meaningful, indicating that mealworm meal supports superior in ovo nutrient availability and embryonic development.

The significant improvements in feed intake, weight gain and livability observed in peafowl chicks supplemented with varying levels of mealworm meal premix are indicative of the nutritional and functional benefits of insect‐based feed ingredients. These results align with prior findings in poultry studies and can be attributed to several interrelated mechanisms involving feed palatability, nutrient density and gut health enhancement (Khan et al. 2023). The increased feed intake, particularly in the early brooding period, is likely influenced by the high palatability and digestibility of mealworm meal. Mealworm meal contains a favourable balance of amino acids, lipids and bioactive compounds that enhance taste and stimulate appetite in young chicks. According to Bovera et al. (2015), inclusion of *T. molitor* larvae in broiler diets led to increased feed intake during the starter phase, attributed to the enhanced palatability and acceptability of insect‐derived proteins. The lipid content—rich in unsaturated fatty acids such as linoleic and oleic acids—may also enhance feed texture and flavour, further encouraging intake (Schiavone et al. 2017).

The improved weight gain in the supplemented groups can be explained by the superior protein quality and high digestibility of insect meals. Mealworm meal provides highly bioavailable essential amino acids—such as methionine and lysine—critical for tissue development and growth in young chicks. These nutrients enhance protein accretion and muscle growth, particularly during the rapid development phase in early life. The sustained weight gain over 4 weeks observed in the present study is comparable to the findings of De Marco et al. (2015), who reported enhanced growth performance in broilers fed diets partially replaced with insect meal.

However, our study did not analyse muscle composition, enzyme activity or gene expression related to growth, which limits our ability to pinpoint the biological pathways responsible for performance improvement.

In addition, insect meal contains antimicrobial peptides and chitin, which may positively influence gut microbiota and immune function (Gasco et al. 2020). We acknowledge that these outcomes were not measured in the present trial; thus, claims regarding immune stimulation or gut modulation remain speculative and should be confirmed by future research incorporating histological and microbial analyses. The improved chick survivability in the mealworm‐supplemented groups—particularly in the first 3 weeks—suggests a functional benefit beyond mere nutritional content. Early chick mortality is often associated with immune suppression, poor gut development and susceptibility to environmental stressors. Insect meals are rich in bioactive compounds such as antimicrobial peptides, lauric acid and chitin derivatives, all of which have been reported to stimulate innate immunity and protect against enteric pathogens. A study by Biasato et al. (2018) demonstrated that the inclusion of insect meal in broiler diets enhanced intestinal morphology and microbial balance, leading to improved health outcomes and reduced mortality. Similar trends have been reported by Loponte et al. (2017), where broiler chicks fed *T. molitor* meal exhibited better survival and resilience to stress during the brooding phase. However, without immune profiling or microbiota analysis, the improved livability observed in this study can only be interpreted as a performance indicator, not as evidence of enhanced immune function. No mortality by Week 4 in all groups further underscores that the initial improvement in growth performance and diet acceptability can establish a stable baseline for survivability once early development challenges are overcome.

## 5 Conclusion

Dietary supplementation with mealworm meal premix improved FCR, growth performance, livability and chick hatch weight in breeding and brooding peafowl, with the most consistent benefits observed at the highest inclusion level (60 mg/kg). However, improvements in fertility and hatchability were not statistically significant and should be interpreted cautiously. Future research should explore the underlying molecular, immunological and histological mechanisms to validate the functional benefits of insect meal supplementation in ornamental bird species.

## Author Contributions

**Sarzamin Khan**: conceptualization. **Hanan Al‐Khalaifah**: funding acquisition. **Haris Khan**: methodology. **Rifat Ullah Khan**: writing – review and editing. **Shabana Naz**: writing – review and editing. **Ala Abudabos**: writing – review and editing. **Ibrahim A. Alhidary**: funding acquisition, resources.

## Ethics Statement

The Committee on Animal Rights and Welfare, the University of Agriculture, Peshawar, Pakistan, approved this study (FAHVS/122/2023).

## Conflicts of Interest

The authors declare no conflicts of interest.

## Data Availability

The data that support the findings of this study are available from the corresponding author upon reasonable request.
